# Use of health systems evidence by policymakers in eastern mediterranean countries: views, practices, and contextual influences

**DOI:** 10.1186/1472-6963-12-200

**Published:** 2012-07-16

**Authors:** Fadi El-Jardali, John N Lavis, Nour Ataya, Diana Jamal, Walid Ammar, Saned Raouf

**Affiliations:** 1 Department of Health Management and Policy, American University of Beirut, Room 107C, PO Box 11–0236, Riad El Solh, Beirut, 1107 2020, Lebanon; 2 McMaster Health Forum, MML-417, 1280 Main St. West, Hamilton, Ontario, L8S 4L6, Canada; 3 Research, Advocacy and Public Policy-making, Issam Fares Institute for Public Policy and International Affairs, American University of Beirut, Box 11–0236, Riad El Solh, Beirut, 1107 2020, Lebanon; 4 McMaster Health Forum, MML-417, 1280 Main St. West, Hamilton, Ontario, L8S 4L6, Canada; 5 Centre for Health Economics and Policy Analysis, McMaster University, CRL-209, 1280 Main St. West, Hamilton, Ontario, L8S 4K1, Canada; 6 Department of Clinical Epidemiology and Biostatistics, McMaster University, CRL-209, 1280 Main St. West, Hamilton, Ontario, L8S 4K1, Canada; 7 Department of Political Science, McMaster University, CRL-209, 1280 Main St. West, Hamilton, Ontario, L8S 4K1, Canada

**Keywords:** Knowledge translation, Eastern Mediterranean region, Health systems research, Evidence- to- policy

## Abstract

**Background:**

Health systems evidence can enhance policymaking and strengthen national health systems. In the Middle East, limited research exists on the use of evidence in the policymaking process. This multi-country study explored policymakers’ views and practices regarding the use of health systems evidence in health policymaking in 10 eastern Mediterranean countries, including factors that influence health policymaking and barriers and facilitators to the use of evidence.

**Methods:**

This study utilized a survey adapted and customized from a similar tool developed in Canada. Health policymakers from 10 countries (Algeria, Bahrain, Jordan, Lebanon Oman, Pakistan, Palestine, Sudan, Tunisia, and Yemen) were surveyed. Descriptive and bi-variate analyses were performed for quantitative questions and thematic analysis was done for qualitative questions.

**Results:**

A total of 237 policymakers completed the survey (56.3% response rate). Governing parties, limited funding for the health sector and donor organizations exerted a strong influence on policymaking processes. Most (88.5%) policymakers reported requesting evidence and 43.1% reported collaborating with researchers. Overall, 40.1% reported that research evidence is not delivered at the right time. Lack of an explicit budget for evidence-informed health policymaking (55.3%), lack of an administrative structure for supporting evidence-informed health policymaking processes (52.6%), and limited value given to research (35.9%) all limited the use of research evidence. Barriers to the use of evidence included lack of research targeting health policy, lack of funding and investments, and political forces. Facilitators included availability of health research and research institutions, qualified researchers, research funding, and easy access to information.

**Conclusions:**

Health policymakers in several countries recognize the importance of using health systems evidence. Study findings are important in light of changes unfolding in some Arab countries and can help undertake an analysis of underlying transformations and their respective health policy implications including the way evidence will be used in policy decisions.

## Background

Health systems evidence can enhance policymaking and strengthen national health systems by identifying priorities, providing broader choice of policy options, informing policy formulation and implementation, and setting the stage for evaluating the outcomes of policies [1-3].

The *Bamako Call to Action,* issued at the Global Ministerial Forum on Research for Health in November 2008, urged national governments and international funders to promote knowledge translation (KT) and exchange through the application of effective and safe interventions, evidence- informed policies, policy- informed research, and effective dissemination of research results for improving health systems performance [4]. This call to action has emphasized the importance of the use of evidence in policymaking and pushed KT to the spotlight in many countries [4]. The *Montreux Statement* at the First Global Symposium on Health Systems Research held in November 2010 reinforced the need for strengthening health systems research and enhancing its translation into policy [5].

One of the primary challenges facing countries in their KT and exchange initiatives is developing effective strategies to promote the use and application of research in policymaking [6]. The literature shows that the success of KT strategies is highly dependent on tailoring the approach to the barriers and facilitators found within a particular and unique setting [6-8]. The available literature attests to the wide gap between the “push” and “pull,” which is also known as the “knowledge- to- action gap”. Reasons for this gap vary across countries and are mainly attributed to lack of interaction between policymakers and researchers, lack of timeliness or relevance of research, mutual mistrust, policymakers’ lack of skills and negative attitudes towards research, political instability, as well as power and budget struggles [1,2,6,9,10].

The application of health systems evidence in policy is a problem faced by many developed and developing countries. However, it is of particular concern in countries where health systems are in a state of rapid transition such as in several Middle Eastern countries. In this region, there are greater policy concerns related to health systems functioning, a wider range of pressing health systems challenges, greater demands on policymakers to be transparent, and an emerging role for non-state actors [3]. Addressing policy concerns and health systems challenges is highly dependent on supporting the use of health systems evidence in policymaking. In the East Mediterranean region (EMR), policymakers recognize the need for evidence for policymaking in health systems and evidence- informed policymaking is now garnering greater attention within the health policy environment [3].

There is a deficiency in health systems research and systematic reviews from the EMR [3,11]. A recent print media analysis in 44 Low and Middle Income Countries (LMICs) which included several countries from the EMR showed that the region is among the lowest in terms of the articles that describe or use health systems research [12]. The EMR has the second lowest proportion (after Africa) of scientific publications addressing health topics in the world (0.8% among all World Health Organization (WHO) regions) [13]. The administrative structures of several countries in the region are crippled by poorly arranged health care systems, which affect health care financing and delivery. This has lead to lack of coordination where several countries face challenges pertaining to poor resource allocation, poor public-private partnerships, and a lack of policies for financial sustainability, which are reflected in poor quality and inefficiency [14]. In a recent priority- setting exercise conducted with policymakers and researchers from the region, participants called for further exploration of health systems research into policy, engaging policymakers in health systems research, and conducting surveys to better understand the policymaking context and design effective KT strategies for the region [3].

In general, most of the evidence base on KT strategies comes from developed countries [1,2,6,9]. Only a few studies explored the use of evidence in policymaking in LMICs [15-19]. The few studies from LMICs show that these countries face a unique set of challenges in promoting KT due to scarce resources (financial and human resources), weakness in health information and delivery, lack of access to research, and corruption [15-23].

In the Middle East region, limited research exists on the use of evidence and contributing factors in the policymaking process [3,19,23-26]. We are not aware of a survey having been conducted about the views and practices of policymakers on the use of health systems evidence in policymaking in the Middle East. The objective of this study is to explore the views and practices of policymakers from 10 Middle Eastern countries regarding the use of health systems evidence in health policymaking. In addition, it aims to elucidate the factors that influence health policymaking and the barriers and facilitators to the use of evidence in the policymaking process. This multi-country study was conducted in 10 Eastern Mediterranean countries selected based on their interest and participation in the launch meeting of the Evidence Informed Policy Network- Eastern Mediterranean Region (EVIPNet EMR), which is a social network that encourages the use of evidence in the policymaking process and includes researchers, policymakers and civil society members from the EMR. Surveyed countries are Algeria, Bahrain, Jordan, Lebanon, Oman, Pakistan, Palestine, Sudan, Tunisia, and Yemen.

## Methods

A cross- sectional survey was distributed to policymakers in 10 countries in the Middle East region from January 2010 to September 2010. Details on sampling of respondents are detailed subsequently herewith. The survey was adapted and customized from a similar tool developed in Canada [27]; some items were rephrased, edited or removed to fit the context of health policymaking in the region. It consisted of a demographics section, two quantitative scales, and open-ended questions. Demographics included questions on age, gender, and degree. The first scale included 14 items that assessed the policymaking context using a five- point scale. The second scale included 16 items on a five- point scale that assessed policymakers’ views and their own practices in the use of evidence in policymaking. The Likert scale was graded on a 5-point likert scale ranging from (in ascending order of scores) strongly agree to agree, neither agree nor disagree, disagree, to strongly disagree. In the open-ended sections, respondents were asked to list the top three factors that exerted the strongest influence in the policymaking process in their respective countries or organizations. They were also asked to provide examples on: (i) the formulation of a policy where evidence was available and had an important role; (ii) where evidence was available but was not used, and (iii) where evidence was not available but was needed. In addition, respondents were also asked to list at least three major barriers and facilitators to the use of evidence in policymaking and three major suggestions to improve the use of evidence in policymaking. Finally, they were asked to list key training needs. The survey was originally developed in English and translated to Arabic by a professional translator. The survey was back-translated to English, minimal differences were detected. It was pilot- tested and further modified to fit the context of the region.

Institutional review board (IRB) approval was obtained prior to data collection from the American University of Beirut (AUB). Focal people in study countries validated lists of potential respondents developed by the research team. They mailed out the surveys (that were placed in sealed envelopes) to the mailing addresses of potential respondents in their countries. They were provided with a detailed procedures manual which included information on how to contact the respondents by telephone or by email, and instructions on how to follow up with respondents, and obtain the completed surveys. Focal people provided respondents with sealed packages that included:

· A sheet with instructions to respondents on how to fill the survey, this included information on how to contact the focal person when the survey was complete. Respondents were also given the option of returning the survey directly to the research team via courier in case they did not want to send it back through the focal person, or faced any difficulty doing so.

· An information sheet about the project including study objectives, briefing on methodology and expected benefits of study findings.

· The survey which included a consent form on the first page. It is worth noting that although the first page of the survey included a consent form, it did not ask respondents to specify or sign their name. Respondents were only asked to tick a box that indicated that they have read and understood the consent form, provide the researchers with permission to use their responses and understand that their names will not be linked to research findings. The consent form reiterated the explanation of the research project and its objectives. The consent form also included information on how to contact the AUB IRB office (email and phone number in addition to the protocol number for the study) in case of any breaches of ethical protocol or even a concern about the survey. Kindly note that no one has contacted AUB IRB regarding any breaches to ethical protocol.

· An empty envelope to return the completed survey was also included. To maintain the anonymity and confidentiality of respondents, they were asked to place the completed survey in the return envelope, seal it then scratch on the seal before returning to the focal people; this information was available in the instruction sheet.

Upon completion of the survey, respondents contacted the focal people to collect the sealed envelopes and return them to the research team in Beirut via the courier that was contracted to do so. Although the research team provided the focal people with the list of potential respondents, they were not asked to specify which of the respondents completed the survey as this would pose a breach of ethics as well. The research team was only provided with the total number of respondents and number of surveys distributed in each study country.

Purposive sampling was conducted. Countries were selected from the Middle East region based on their interest and participation in the launch meeting of the EVIPNet EMR that took place in January 2009. EVIPNet EMR members were asked to act as focal persons for this study and coordinate activities at the country level including selection of respondents and facilitating the administration of the survey.

A sampling frame was established to determine the selection criteria for respondents from these countries. The sampling frame was based on a similar tool developed in Canada [27]. The sampling frame included policymakers from different settings, defined as those who are responsible for health policy decisions, strategy and planning, at the national level, such as senior officials from the ministry of health (MOH) and other health- related ministries, as well as managers in non- governmental organizations (NGOs), professional associations, and donor agencies. The sampling frame included four main role categories and the descriptive positions corresponding to each of these categories as well as specific examples on these positions. The first category was directors in the MOH and the corresponding positions for this category included: minister of health or deputy minister/ undersecretary/ secretary general, advisors and members of councils, as well as heads of financial and administrative affairs, policy and planning, human resources, pharmaceuticals and laboratories, primary healthcare, public health program, and infectious disease program. The second category was directors in other health- related ministries such as the ministry of education, ministry of finance, and ministry of labor. The third category was managers in NGOs and the corresponding descriptive positions included: most senior managers/ directors in international and national NGOs. The fourth category was staff/ members of a health professional association and the corresponding positions included: most senior managers/ directors in national medical associations, national nursing associations, national pharmacists' associations, and national hospital associations. A similar sampling frame was used for identifying policymakers and stakeholders from the region in other studies [3,28]. The names and contact information of policymakers and stakeholders who fit these specific categories and positions were retrieved and aggregated by the research team based on a thorough internet search of the Ministry of Health websites, professional associations, NGOs, and donor agencies. The aggregated sampling frame for each country was subsequently sent to the corresponding focal person for validation and completion of missing information and positions. Focal people were then requested to send surveys to the policymakers and stakeholders on the validated sampling frame. A procedures manual was developed and distributed to focal people to ensure standardized data collection across countries. Respondents were asked to complete the survey and return it to the research team within two weeks. Respondents who had not completed the survey within allotted time were contacted twice for follow- up; once after two weeks of receiving the survey and again a week after the last contact. To obtain a holistic perspective of the health policymaking process and the role of evidence in policymaking in the region, a target was initially set to survey around 25 policymakers from each country.

Data was entered using CSPro 4.0 and analyzed using the Statistical Package for Social Sciences (SPSS) 19.0 (significance level of 0.05). Descriptive analysis was performed for closed-ended questions (including demographics). The uppermost and lowermost ends of the scales were combined for the purpose of descriptive analysis i.e., strongly agree with agree and strongly disagree with disagree. Histograms indicated that all items were normally distributed. A one-sample t-test was used to compare responses in each country to the overall item mean. Moreover, t-test and ANOVA using Bonferroni multiple F contrast was used to compare responses by respondent affiliation. To compare respondents, they were first broken down into two groups: Government affiliated (i.e. respondents from the MOH) and non-Government affiliated (i.e. respondents from NGOs, professional associations, and donor agencies). These two groups were analyzed using an independent sample t-test. Respondents in non-Government affiliated organizations were analyzed separately as well to account for the specificities pertaining to each of the three settings. These groups were analyzed using ANOVA.

Thematic analysis was used for the qualitative component of the survey. Responses were broken into similar concepts and ideas (open coding). Axial coding followed in which concepts were organized into themes [29]. Recurring themes and emerging patterns across respondents from study countries were then analyzed. Analysis was conducted by two members of the research team with a high level of agreement between the two. Disagreements were discussed until consensus was achieved.

## Results

A total of 421 policymakers were requested to complete the survey, of these, 237 responded (response rate 56.3%). Overall, 51.7% of respondents indicated having a master’s degree. Many respondents (68.8%) worked as policymakers in the MOH while 15.8% worked in NGOs, 8.1% in professional associations, and 8% in donor agencies (Table 1). A total of 62.6% of respondents indicated that they have had training in health policy (Table 1).

**Table 1:** Characteristics of respondents (N = 237)

**Country where respondents work as policymakers**	**N (%)**	**Questionnaires Sent**	**Response Rate**
Pakistan	42 (17.7%)	50	84.0%
Sudan	29 (12.2%)	37	78.4%
Palestine	28 (11.8%)	37	75.7%
Jordan	27 (11.4%)	34	79.4%
Yemen	27 (11.4%)	36	75.0%
Oman	23 (9.7%)	49	46.9%
Algeria	22 (9.3%)	49	44.9%
Lebanon	20 (8.4%)	24	83.3%
Bahrain	10 (4.2%)	55	18.2%
Tunisia	9 (3.8%)	50	18.0%
Total	237	421	56.3%
**Respondents degrees (they were allowed to indicate more than 1 degree)**			
MS/MA/MBA/MPH/Med MSc	122 (51.7%)		
MD or similar	99 (41.8%)		
BS/BA/BSN	52 (21.9%)		
PhD or DPH	40 (16.9%)		
other degree	11 (4.6%)		
**Respondents’ main domain of work**			
MOH	161 (68.8%)		
NGOs	37 (15.8%)		
Professional associations	19 (8.1%)		
Donor agencies	19 (8.1%)		
**Respondents received training in health policy**			
Yes	144 (62.6%)		
No	86 (37.4%)		

### Factors that influence health policymaking

The majority of policymakers (74.9%) reported that lack of coordination across different ministries and between the government and health providers hindered the health policymaking process (65.2%). Overall, 79.2% of respondents reported that limited public funding for the health sector, and the agendas of donor organizations (72.5%), values of the governing parties (53.9%) and research about problems related to healthcare or health systems (45.5%) exerted a strong influence on the policymaking process (Table 2).

**Table 2:** Factors that influence health policymaking and views and practices on the use of evidence in the region

	**Strongly Disagree/Disagree**	**Neither Agree nor Disagree**	**Strongly Agree/Agree**
**Factors that Influence Health Policymaking**			
1-Lack of coordination in governmental/ministerial relations across different ministries (such as the Ministry of Health, Ministry of Finance, etc.) hindered the health policymaking process.	31 (13.2%)	28 (11.9%)	176 (74.9%)
2-Lack of coordination in government/ health provider relations hindered the health policymaking process.	44 (18.9%)	37 (15.9%)	152 (65.2%)
3-Physician associations exerted a strong influence on the health policymaking process.	117 (50.4%)	56 (24.1%)	59 (25.4%)
4-Nursing associations exerted a strong influence on the health policymaking process.	146 (63.8%)	63 (27.5%)	20 (8.7%)
5-Other types of health professional associations exerted a strong influence on the health policymaking process (e.g., Syndicate of hospitals).	115 (49.4%)	66 (28.3%)	52 (22.3%)
6-Private health providers exerted a strong influence on the health policymaking process.	91 (39.6%)	61 (26.5%)	78 (33.9%)
7-Private insurers exerted a strong influence on the health policymaking process.	120 (54.8%)	62 (28.3%)	37 (16.9%)
8-Values of governing parties exerted a strong influence on the health policymaking process.	58 (25%)	49 (21.1%)	125 (53.9%)
9-Public opinion exerted a strong influence on the health policymaking process.	78 (33.2%)	64 (27.2%)	93 (39.6%)
10-Media exerted a strong influence on the health policymaking process.	56 (23.8%)	81 (34.5%)	98 (41.7%)
11-Research about problems related to healthcare or health systems exerted a strong influence on the health policymaking process.	74 (31.5%)	54 (23%)	107 (45.5%)
12-Limited public funding for health exerted a strong influence on the health policymaking process.	31 (13.1%)	18 (7.6%)	187 (79.2%)
13-Other countries’ health policies exerted a strong influence on the health policymaking process.	60 (25.6%)	78 (33.3%)	96 (41%)
14-Donor organizations (e.g., United States Agency for International Development (USAID), United Nations, World Bank, World Health Organization (WHO)) exerted a strong influence on the health policymaking process.	25 (10.6%)	40 (16.9%)	171 (72.5%)
**Views and practices on the use of evidence**			
1-I generally look and/or ask for scientific evidence to support my work in formulating and implementing health policies.	9 (3.8%)	18 (7.7%)	208 (88.5%)
2-I have access to health research through an internet connection at my organization.	29 (12.3%)	21 (8.9%)	185 (78.7%)
3-There are contact and collaborative relations between researchers and health policymakers/ decision makers in my organization.	68 (29.3%)	64 (27.6%)	100 (43.1%)
4-I participated in meetings with researchers to identify high-priority policy issues for which research is needed to inform how to address these issues.	34 (14.5%)	37 (15.8%)	163 (69.7%)
5-Health policymakers request scientific evidence in the policymaking process.	66 (28.1%)	55 (23.4%)	114 (48.5%)
6-The scientific evidence is delivered at the right time.	99 (42.9%)	68 (29.4%)	64 (27.7%)
7-There are summaries of evidence with messages that specify possible actions about health policies issues I confronted in my organization.	83 (35.5%)	69 (29.5%)	82 (35.0%)
8-The available scientific evidence provides sufficient information on the impacts, costs and concrete benefits of the studied or soon-to-implement health policies.	75 (32.2%)	67 (28.8%)	91 (39.1%)
9-The available scientific evidence is delivered with information about its quality and local applicability.	93 (40.1%)	67 (28.9%)	72 (31.0%)
10-There is a sufficient quantity of health research that may contribute to inform the health policymaking/decision making process.	85 (36.8%)	50 (21.6%)	96 (41.6%)
11-There are clearly identified places to find or to ask for scientific evidence that may inform the health policymaking/decision making process.	78 (33.3%)	44 (18.8%)	112 (47.9%)
12-Health policymakers use scientific evidence in the policymaking process whenever it is available and supplied to them.	56 (23.9%)	56 (23.9%)	122 (52.1%)
13-I have received training to acquire, assess the quality and local applicability of scientific evidence, and apply scientific evidence in health policymaking/decision making.	76 (32.5%)	34 (14.5%)	124 (53%)
14-There is explicit budget or funding for both research and evidence- informed health policymaking within my organization.	130 (55.3%)	47 (20%)	58 (24.7%)
15-There is an administrative structure suitable to support an evidence- informed health policymaking process (for example; a policy analysis department or a decision support unit, or the availability of resources, incentives and time for the use of scientific evidence in health policymaking).	123 (52.6%)	45 (19.2%)	66 (28.2%)
16-The political actors related with health (political parties, ministers, parliament, other ministries, etc.) value the use of scientific evidence in the policymaking process.	84 (35.9%)	83 (35.5%)	67 (28.6%)

Policymakers’ responses to an open- ended question further confirmed that health funding and resources, values of governing political parties and political interests of policymakers, and donor organizations and international organizations (e.g., WHO and UNICEF), as well as availability of both national and international research are the top factors that influenced health policymaking processes in the region.

### Policymakers’ views and practices on the use of evidence

Most respondents (88.5%) reported looking or asking for scientific evidence to support the formulation and implementation of health policies. Around half of the respondents (53%) reported receiving training to acquire scientific evidence, assess its quality and local applicability, and apply it in health policymaking. Many respondents (69.7%) indicated participating with researchers to identify high-priority policy issues for which research is needed and less than half of them (43.1%) reported that contact and collaborative relations between researchers and health policymakers exist in their respective organizations (Table 2).

Most respondents (78.7%) indicated that they have access to health research through an internet connection at their respective organizations. Less than half of the respondents (47.9%) reported that there are clearly identified places to find or to ask for scientific evidence, and 41.6% stated that there is sufficient quantity of health research and that it provides sufficient information on the impacts and costs of policies (39.1%). Less than half of the respondents (42.9%) reported that research is not delivered at the right time, lacks information about its quality and local applicability (40.1%), and lacks actionable messages (35.5%) (Table 2).

Around half of the respondents (52.1%) indicated using evidence whenever it is available and supplied to them while 48.5% specifically request it for the policymaking process. More than half of respondents (55.3%) indicated that support for evidence- informed policymaking in their respective countries or organizations was limited due to lack of an explicit budget for evidence- informed health policymaking, and lack of an administrative structure (52.6%). Moreover, 35.9% of respondents reported that limited value is given to evidence by political actors (Table 2).

### Comparing responses by study countries

Below are the results of the one-sample t-test to compare country means to overall item means. Items comprise two scales, the first on factors influencing the policymaking process, and the second on the use of evidence in the policymaking process.

#### Factors influencing policymaking process

Results of the comparison of means with regard to factors influencing the policymaking process are detailed in Table 3. Compared to other countries, Yemen was found to face several challenges with the factors influencing the health policymaking process. One such challenge was lack of coordination in government/ministerial relations across different ministries. In addition, physician, nursing and other associations did not appear to have significant influence on the health policymaking process as compared to other countries. The influence of private providers and insurers on the policymaking process was also minimal (Table 3).

**Table 3:** Comparing mean differences in factors influencing the health policymaking process and use of evidence in the policymaking across the study countries (* indicates p-values <0.05, ** indicates p-values <0.001)

	**Overall**	**Lebanon**	**Pakistan**	**Palestine**	**Jordan**	**Bahrain**	**Sudan**	**Yemen**	**Oman**	**Algeria**	**Tunisia**
	**N = 237**	**N = 20**	**N = 42**	**N = 28**	**N = 27**	**N = 10**	**N = 29**	**N = 27**	**N = 23**	**N = 22**	**N = 9**
	**Mean (SD)**	**Mean (SD)**	**Mean (SD)**	**Mean (SD)**	**Mean (SD)**	**Mean (SD)**	**Mean (SD)**	**Mean (SD)**	**Mean (SD)**	**Mean (SD)**	**Mean (SD)**
**Factors influencing the health policymaking process**											
1-Lack of coordination in governmental/ministerial relations across different ministries (such as the Ministry of Health, Ministry of Finance, etc.) hindered the health policymaking process.	3.86 (1.02)	4.20 (0.70)	3.93 (0.95)	3.79 (0.92)	4.04 (0.90)	3.2 (1.62)	4.24 (0.87)*	4.27 (0.78)*	3.09 (1.24)*	3.5 (0.96)	3.56 (1.01)
2-Lack of coordination in government/ health provider relations hindered the health policymaking process.	3.62 (1.01)	3.55 (0.83)	3.76 (0.97)	3.82 (0.86)	4.04 (0.82)*	3.20 (1.40)	3.62 (1.12)	3.85 (0.78)	3.04 (1.19)*	3.45 (1.06)	3.00 (1.00)
3-Physician associations exerted a strong influence on the health policymaking process.	2.6 (1.17)	3.35 (1.14)	2.88 (1.10)	2.29 (0.94)	3 (1.07)	2.78 (0.97)	2.48 (1.30)	1.63 (0.79)**	2.14 (1.11)	2.68 (1.25)	3.33 (0.87)*
4-Nursing associations exerted a strong influence on the health policymaking process.	2.22 (0.91)	2.25 (0.79)	2.26 (0.83)	2.18 (0.82)	2.56 (0.85)*	2.67 (0.71)	2.14 (0.95)	1.46 (0.71)**	2.15 (1.09)	2.43 (0.98)	2.88 (0.84)
5-Other types of health professional associations exerted a strong influence on the health policymaking process (e.g., Syndicate of hospitals).	2.59 (1.07)	3.70 (0.80)**	2.67 (0.85)	2.11 (0.88)**	3 (1.18)	2.60 (0.70)	2.34 (0.90)	1.65 (0.85)**	2.19 (1.03)	3.14 (1.08)*	3.11 (0.93)
6-Private health providers exerted a strong influence on the health policymaking process.	2.87 (1.08)	3.60 (1.10)**	2.67 (1.03)	2.96 (1.14)	3.3 (1.03)*	3.30 (0.82)	2.96 (1.02)	2.37 (1.04)*	2.3 (0.88)*	2.71 (1.01)	3.00 (1.00)
7-Private insurers exerted a strong influence on the health policymaking process.	2.42 (1.06)	2.80 (0.95)	2.45 (0.92)	2.32 (0.91)	2.44 (1.03)	2.56 (0.88)	3.1 (1.29)*	1.92 (1.02)*	1.96 (0.98)*	2.09 (1.14)	2.33 (0.87)
8-Values of governing parties exerted a strong influence on the health policymaking process.	3.36 (1.22)	3.65 (1.39)	3.15 (1.04)	3.36 (1.22)	2.67 (1.8)*	3.70 (0.82)	4.00 (1.10)*	3.69 (1.09)	2.43 (1.29)*	3.5 (1.10)	4.22 (0.67)*
9-Public opinion exerted a strong influence on the health policymaking process.	3.04 (1.09)	2.80 (1.20)	2.67 (1.00)**	2.71 (1.08)	3.33 (1.11)	3.7 (0.82)*	3.34 (1.11)	2.81 (0.98)	3.43 (0.99)	3.00 (1.16)	3.44 (0.88)
10-Media exerted a strong influence on the health policymaking process.	3.21 (0.99)	3.40 (0.94)	2.95 (0.99)	2.79 (0.83)	3.67 (0.96)*	3.5 (1.18)	3.57 (1.10)	3.11 (0.93)	3.13 (1.14)	3.14 (0.77)	3.22 (0.67)
11-Research about problems related to healthcare or health systems exerted a strong influence on the health policymaking process.	3.17 (1.13)	3.30 (0.92)	3.12 (1.13)	2.79 (1.20)	2.96 (1.13)	3.5 (0.97)	3.54 (1.04)	2.81 (1.15)	3.78 (1.09)*	2.82 (1.18)	3.78 (0.83)
12-Limited public funding for the health sector exerted a strong influence on the health policymaking process.	4.05 (1.10)	4.25 (0.91)	3.81 (1.38)	4.21 (0.79)	3.63 (1.25)	4.3 (1.25)	4.52 (0.68)*	4.3 (0.95)	3.78 (1.20)	3.86 (1.13)	4.00 (0.87)
13-Other countries’ health policies exerted a strong influence on the health policymaking process.	3.14 (1.00)	2.85 (0.99)	2.76 (0.87)**	3.54 (0.92)	3.37 (0.74)	3.2 (1.03)	3.21 (1.15)	2.96 (0.96)	3.61 (0.94)*	2.95 (1.24)	3.00 (0.87)
14-Donor organizations (e.g., United States Agency for International Development (USAID), United Nations, World Bank, World Health Organization (WHO)) exerted a strong influence on the health policymaking process.	3.83 (1.01)	4.00 (0.97)	3.86 (0.84)	4.32 (0.82)	4.00 (1.04)	3.2 (0.92)	4.07 (0.84)	4.04 (0.90)	3.43 (1.16)	3.14 (1.17)*	3.22 (1.09)
**Use of evidence in the health policymaking process**											
1-I generally look and/or ask for scientific evidence to support my work in formulating and implementing health policies.	4.28 (0.80)	4.70 (0.47)*	4.2 (0.93)	4.36 (0.68)	4.04 (0.76)	4.5 (0.53)	4.52 (0.51)*	3.96 (1.02)	4.48 (0.67)	3.95 (0.95)	4.33 (0.71)
2-I have access to health research through an internet connection at my organization.	3.94 (1.06)	3.75 (1.21)	3.98 (1.28)	4.18 (0.77)	3.81 (1.04)	4.4 (0.70)	4.21 (0.86)	3.63 (1.15)	3.74 (1.14)	3.68 (1.08)	4.44 (0.53)*
3-There are contact and collaborative relations between researchers and health policymakers/ decision makers in my organization.	3.15 (1.07)	3.45 (1.15)	3.17 (1.14)	3.39 (0.99)	2.74 (0.98)*	3.8 (0.63)*	3.14 (0.89)	2.63 (1.15)*	3.3 (1.11)	3.1 (1.07)	3.44 (1.13)
4-I participated in meetings with researchers to identify high-priority policy issues for which research is needed to inform how to address these issues.	3.71 (1.06)	4.15 (0.49)*	3.63 (1.22)	3.82 (0.86)	3.37 (1.18)	4 (0.82)	4.07 (0.96)	3.31 (1.16)	3.65 (1.23)	3.41 (0.96)	4.44 (0.53)*
5-Health policymakers request scientific evidence in the policymaking process.	3.26 (1.09)	3.75 (1.07)	3.22 (1.13)	3.07 (1.09)	2.81 (1.11)*	4.2 (0.63)*	3.07 (1.00)	2.93 (1.21)	3.35 (0.89)	3.50 (1.01)	4.00 (0.87)*
6-The scientific evidence is delivered at the right time.	2.77 (1.01)	2.7 (0.92)	2.9 (1.06)	2.75 (0.80)	2.56 (0.89)	3.6 (0.52)*	2.48 (0.98)	1.85 (0.77)**	3.00 (0.91)	3.52 (1.03)*	3.33 (1.00)
7-There are summaries of evidence with messages that specify possible actions about health policies issues I confronted in my organization.	2.96 (1.08)	3.1 (1.07)	3 (1.13)	3.14 (1.01)	2.7 (0.91)	3.1 (0.99)	2.93 (1.00)	2.41 (1.19)*	3.09 (1.20)	3.41 (1.01)*	2.89 (1.05)
8-The available scientific evidence provides sufficient information on the impacts, costs and concrete benefits of the studied or soon-to-implement health policies.	3.04 (1.05)	3.15 (1.04)	2.83 (1.16)	3.00 (1.05)	2.69 (0.88)	3.6 (0.70)*	3.07 (1.10)	2.67 (1.07)	3 (1.02)	3.64 (0.79)*	3.89 (0.60)*
9-The available scientific evidence is delivered with information about its quality and local applicability.	2.87 (1.05)	3.2 (1.06.)	2.83 (1.05)	2.96 (1.11)	2.62 (0.94)	3.5 (0.71)*	2.69 (1.11)	2.22 (0.97)*	2.95 (0.95)	3.23 (0.92)	3.63 (0.92)
10-There is a sufficient quantity of health research that may contribute to inform the health policymaking/decision making process.	3.02 (1.14)	3.25 (1.12)	3.05 (1.16)	3.04 (1.09)	2.58 (1.14)	3.1 (1.10)	3.1 (1.08)	2.68 (1.25)	2.78 (1.04)	3.5 (0.96)*	3.56 (1.33)
11-There are clearly identified places to find or to ask for scientific evidence that may inform the health policymaking/decision making process.	3.15 (1.09)	3.7 (0.92)*	3.24 (1.14)	3.21 (0.92)	2.69 (1.16)	3.9 (0.74)*	3.07 (1.03)	2.56 (0.89)*	2.7 (1.19)	3.64 (1.00)*	3.78 (0.67)*
12-Health policymakers use scientific evidence in the policymaking process whenever it is available and supplied to them.	3.29 (1.02)	3.3 (1.17)	3.17 (1.18)	2.86 (1.08)*	3.00 (0.98)	3.9 (0.32)**	3.59 (0.83)	3.11 (1.05)	3.96 (0.64)**	3.23 (0.81)	3.44 (1.01)
13-I have received training to acquire, assess the quality and local applicability of scientific evidence, and apply scientific evidence in health policymaking/decision making.	3.26 (1.28)	3.45 (1.40)	3.22 (1.17)	3.50 (1.00)	2.96 (1.37)	3.6 (1.43)	3.54 (1.32)	2.81 (1.42)	3 (1.35)	3.64 (1.09)	3 (1.32)
14-There is explicit budget or funding for both research and evidence- informed health policymaking within my organization.	2.54 (1.13)	2.55 (1.19)	2.61 (1.16)	2.43 (1.17)	2.15 (0.86)*	2.9 (0.99)	2.34 (0.94)	2.44 (1.42)	2.7 (1.02)	3.05 (1.17)	2.67 (1.12)
15-There is an administrative structure suitable to support an evidence- informed health policymaking process (for example; a policy analysis department or a decision support unit, or the availability of resources, incentives and time for the use of scientific evidence in health policymaking).	2.62 (1.18)	2.45 (1.00)	3.18 (1.26)*	2.54 (1.11)	2 (0.83)*	3.3 (1.06)	2.66 (1.05)	2.33 (1.24)	2.26 (1.25)	2.86 (1.28)	2.89 (0.93)
16-The political actors related with health (political parties, ministers, parliament, other ministries, etc.) value the use of scientific evidence in the policymaking process.	2.81 (1.11)	2.5 (1.15)	2.78 (1.39)	2.82 (1.25)	2.85 (0.99)	3.1 (0.57)	2.76 (1.15)	2.56 (0.97)	3.14 (0.94)	2.73 (0.93)	3.56 (0.73)*

Jordan is another country facing slightly similar challenges in the factors influencing the policymaking process. However, the challenge of lack of coordination was more in government/health provider relations. While nursing associations also had a poor influence on the policymaking process in Jordan, it was significantly better as compared to the overall mean and to other countries as well. While results showed that Jordan fared slightly better than Oman with regard to the influence of the values of governing parties on the policymaking process, this influence was still not as highly pronounced as compared to Sudan. However, the influence of the media on the policymaking was significantly better than the overall mean and in relation to other countries (Table 3).

If we also consider the case of Oman, the lack of coordination in government and ministerial and health provider relations did not pose as much of a challenge as compared other countries. The influence of private providers and values governing parties also didn’t appear to pose a challenge for Oman as compared to other countries. However, the influence of research about problems related to healthcare did appear to have a stronger influence on the health policymaking process in Oman as compared to the overall mean and other countries as well (Table 3).

#### Use of evidence in the health policymaking process

Results of the comparison of means on items describing the use of evidence in the policymaking process are detailed in Table 3. Yemen also emerged as a country facing challenges with use of evidence in the policymaking process. Specifically, and compared to other countries, policymakers in Yemen reported having much fewer contacts and collaboration between researchers and health policymakers. Moreover, in Yemen, scientific evidence is not delivered at the right time and lacks information on quality and local applicability. Additionally, summaries of evidence with messages specifying possible actions are not as readily available compared to other countries. Yemen also scored low on having clearly identified places to find or ask for scientific evidence to inform the policymaking process (Table 3).

The case of Bahrain is an exact opposite of that of Yemen. For instance, Bahrain scored highest compared to other countries when it came to having contacts and collaborative relations between policymakers and researchers. In Bahrain, scientific evidence is reportedly delivered at the right time. In fact, Bahrain had the highest score when it came to having policymakers request scientific evidence for the policymaking process and also using evidence whenever it is available and supplied to them. Bahrain also had the highest score on delivery of scientific evidence with information about quality and local applicability and also when it came to having clearly identified places to find or ask for evidence that may inform the policymaking process. It was also reported that the available evidence in Bahrain provides sufficient information about impacts, costs and benefits of soon-to-implement health policies (Table 3).

Tunisia also fared slightly better on use of evidence in policymaking as compared to other countries. If we compare Tunisia to other countries, it had the highest mean score on access to health research through an internet connection. Tunisia also had the highest score on participation in meetings with researchers to identify high-priority policy issues for which evidence is needed. It is worth noting that Tunisia scored lower than Bahrain when it came to policymakers requesting evidence for the policymaking process and having clearly identified places to ask for evidence. However, Tunisia scored better than Bahrain on the item assessing whether the available evidence in Bahrain provides sufficient information about impacts, costs and benefits of soon-to-implement health policies. Tunisia also fared better than other countries with regard to having political actors who value the use of scientific evidence in the policymaking process (Table 3).

### Comparing responses by respondent affiliation

Respondents were broken down into two groups, government affiliated (i.e. those working at the MOH) and non-Government affiliated policymakers (i.e. NGOs, professional associations and donor agencies). Differences between these two groups were assessed using a t-test. Respondents working in the three non-Government affiliated organizations were then compared to respondents working at the MOH using ANOVA using Bonferroni multiple F contrast. Only statistically significant items are reported in Table 4.

**Table 4:** Comparing responses by respondent affiliation

		**Government Affiliated**		**Non-Government affiliated**							
		**MOH**		**Total**^†^		**NGO**		**Professional Association**		**Donor Agency**	
		**N = 161**		**N = 75**		**N = 37**		**N = 19**		**N = 19**	
		**Mean**	**SD**	**Mean**	**SD**	**Mean**	**SD**	**Mean**	**SD**	**Mean**	**SD**
Lack of coordination in governmental/ministerial relations across different ministries (such as the Ministry of Health, Ministry of Finance, etc.) hindered the health policymaking process.	^1, 2^	3.72	1.11	4.23	0.70	4.33	0.62	4.22	0.67	4.00	0.894
Lack of coordination in government/ health provider relations hindered the health policymaking process.	^1, 2^	3.46	1.07	4.09	0.78	4.04	0.85	4.33	0.71	4.00	0.632
Private insurers exerted a strong influence on the health policymaking process.	^1, 2, 3^	2.29	1.07	3.00	0.88	2.88	0.91	3.38	0.74	3.00	0.894
Public opinion exerted a strong influence on the health policymaking process.	^2^	3.18	1.03	2.55	1.14	2.41	1.15	2.89	1.27	2.64	1.027
Limited health funding exerted a strong influence on the health policymaking process.	^4^	4.09	1.11	3.98	1.07	4.33	0.78	3.78	1.20	3.27	1.272
There are contact and collaborative relations between researchers and health policymakers/decision makers in my organization.	^2^	3.01	1.08	3.53	0.98	3.74	0.98	3.67	0.71	2.91	0.944
Health policymakers request scientific evidence in the policymaking process.	^5^	3.29	1.08	3.19	1.08	3.19	1.00	4.11	0.93	2.45	0.82
The available scientific evidence provides sufficient information on the impacts, costs and concrete benefits of the studied or soon-to-implement health policies.	^5^	3.08	1.00	3.15	1.07	3.27	1.12	3.67	0.71	2.45	0.934
There is a sufficient quantity of health research that may contribute to inform the health policymaking/decision making process.	^3^	2.94	1.12	3.36	1.21	3.24	1.23	4	0.71	3.09	1.375
Health policymakers use scientific evidence in the policymaking process whenever it is available and supplied to them.	^2^	3.48	0.92	2.83	1.12	3.08	1.13	2.44	1.01	2.55	1.128
The political actors related with health (political parties, ministers, parliament, other ministries, etc.) value the use of scientific evidence in the policymaking process.	^1^	2.96	1.04	2.55	1.19	2.70	1.30	2.33	1.11	2.36	1.03

^†^ This column refers to the overall mean for Non-Government affiliated respondents. The T-test compares values in this column to that in the one with the heading “Government affiliated – MOH”.

^1^ significant difference between Government affiliated (MOH) and non-Government affiliated (Total).

^2^ significant difference between MOH and NGO.

^3^ significant difference between MOH and Professional Association.

^4^ significant difference between NGO and Donor agencies.

^5^ significant difference between Professional Association and Donor agencies.

Significant differences in responses on factors influencing health policy formulation were observed between respondents working in different areas. Results showed that respondents working in non-Government affiliated organizations had significantly higher agreement scores than respondents working in the MOH regarding the lack of coordination across different ministries and health providers. Specifically, respondents from NGOs had higher agreement scores when it came to these two items compared to those from the MOH. Respondents working in non-Government affiliated organizations perceived greater pressure by private insurers on the health policymaking process than their counterparts in the MOH with higher agreement scores observed for respondents from professional associations and NGOs (Table 4). Public opinion, on the other hand, was perceived to have a greater influence on the health policymaking process by respondents in the MOH compared respondents from NGOs (Table 4). Significantly higher agreement scores were also observed for respondents from NGOs compared to those from donor agencies regarding the influence of limited funding on the health policymaking process (Table 4).

Significant differences were also observed between respondents with regard to the use of evidence in health policymaking. Respondents in NGOs reported significantly higher means regarding the degree of contact and collaboration between researchers and policymakers. On the other hand, respondents in the professional associations reported higher agreement scores on the degree to which policymakers request scientific evidence for the policymaking process than those working in Donor agencies (Table 4). Donor agencies reported significantly lower agreement scores than professional associations when it came to whether the existing evidence provides sufficient information for soon-to-implement policies (Table 4). Moreover, respondents from non-Government affiliated organizations had significantly lower scores than those in professional associations when asked whether there is a sufficient quantity of health research to inform the policymaking. Respondents from NGOs had significantly lower scores when asked whether policymakers used evidence when it was made available to them in the policymaking process. When asked whether political actors value the use of scientific evidence, respondents from the MOH had significantly higher agreement scores than their counterparts (Table 4).

### Examples on health policymaking in the MENA region

#### Formulation of a health policy where evidence was available and used

In response to an open- ended question, the most frequently mentioned examples of evidence being used in the formulation of policies are: maternal and child health (10%) and health strategic plan (10%). Some respondents also cited vaccination schedules and accreditation strategies as examples of health policies where evidence was used (Table 5).

**Table 5:** Examples of a health policy where evidence was available and had an important role, where evidence was available but was not used, and where evidence was not available but was needed

**Health policy where evidence was available and had an important role (n = 194)**		**Health policy where evidence was available but was not used (n = 154)**		**Health policy where evidence was not available but was needed (n = 141)**	
·Maternal and child health	20 (10%)	·Human resources strategies and needs and evaluation of training programs	15 (10%)	·Implementation of chronic disease screening and prevention programs	13 (9%)
·Health strategic plan	19 (10%)	·Chronic disease screening, prevention, and treatment	15 (10%)	·Implementation of accreditation and quality improvement plans	12 (9%)
·Vaccination guidelines and schedules	18 (9%)	·Starting new medical centers, programs and services	13 (8%)	·Health strategic plan	12 (9%)
·Strategies on accreditation and healthcare quality improvement	12 (6%)			·Distribution of healthcare workers	11 (8%)

n = total number of responses to each question.

#### Formulation of a health policy where evidence was available but not used

Respondents most frequently cited existing health human resources strategies and needs in addition to the evaluation of healthcare professionals’ training programs (10%) as examples of policies not formulated using available evidence. For example, a policymaker from Lebanon stated that “No policy [was formulated] at the educational level to control the high number of physicians due to political reasons” and another policymaker from Pakistan stated that “population age and sex structure data is not being used for developing human resources strategies”. Furthermore, respondents frequently reported that certain policies on chronic disease screening, prevention and treatment were formulated without using available evidence (9.7%). Respondents also frequently indicated that the establishment of new healthcare centers was not based on feasibility studies and needs assessment. For instance, a policymaker from Jordan reported that “[the decision to] establish new health centers was influenced mainly by politicians” (Table 5).

#### Formulation of a health policy where evidence was needed but not available across countries

In response to this open- ended question, respondents most commonly indicated that evidence was needed on how to locally apply programs related to chronic diseases (9%) and accreditation (9%). Policymakers also indicated that evidence is needed to substantiate national health strategic plans, as a policymaker from Yemen stated “review and evaluation of the health system based on [evidence from] country specific health indicators [is needed]”. Another policymaker from Palestine stated that “evaluation of financial policies and the allocation of resources, and their impact on the health of the population [is needed]”. They also reported that evidence was needed to formulate policies on the distribution of healthcare workers. A policymaker from Jordan stated that more local evidence is needed for the formulation of “a policy that requires hospitals to apply accreditation standards to improve their health care services” and another policymaker from Lebanon stated that “[locally applicable] patient satisfaction surveys [are needed] for accreditation” (Table 5).

### Barriers and facilitators to evidence- informed policies and strategies to improve evidence to policy

In response to this question, respondents most frequently reported lack of funding and investments in priority health research and in implementing evidence from research in policy (16%) and lack of policy relevant research (14%) as barriers to evidence- informed policies. Other commonly mentioned barriers are over- riding political forces, corruption, and weak administrative structure of policymaking, as well as lack of trained policymakers in accessing and using evidence (Table 6).

**Table 6:** Barriers and facilitators to evidence- informed policies and strategies to improve evidence to policy

**Barriers to evidence- informed policies (n = 446)**		**Facilitators to evidence- informed policies (n = 145)**		**Strategies to improve evidence to policy (n = 570)**	
·Lack of funding and investment in priority health research and in implementing evidence from research in policy	69 (16%)	·Availability of policy relevant health research	22 (15%)	·Build the capacity of policymakers in locating proper information, assessing the quality of research, its cost effectiveness and local applicability	75 (13%)
·Lack of policy relevant research	62 (14%)	·Easy access to information	16 (11%)	·Increase funding and investments in health research	7 (13%)
·Over- riding political forces	49 (11%)	·Availability of research funding	13 (9%)	·Improve dissemination and translation of research	59 (10%)
·Lack of political will, corruption, and weak administrative structure of policy making entities	49 (11%)	·Support of NGOs and international organizations that drive the use of research in policymaking	13 (9%)	·Conduct health systems research to inform policy	44 (8%)
·Lack of trained policy makers in accessing and using evidence for policy making	43 (10%)	·Availability of research centers	11 (8%)	·Establish evidence- to- policy decision support unit that supports policy makers in using research in policy.	41 (7%)
		·Belief of policymakers in the importance of evidence	11 (8%)	·Conduct sensitization and awareness workshops on evidence- informed policymaking	40 (7%)
		·Availability of qualified researchers	10 (7%)	·Improving contact and exchange between policymakers and researchers	31 (5%)
		·Communication and networking between policymakers and researchers	10 (7%)		
		·Wide dissemination of research	10 (7%)		
		·Qualified policymakers	10 (7%)		

n = total number of responses to each question, respondents listed up to three responses.

Commonly reported facilitators to evidence- informed policies in the region include availability of policy relevant health research (15%), easy access to information (11%), availability of research funding (9%), support of NGOs and international organizations (9%). Other frequently mentioned facilitators are availability of research centers, policymakers’ belief in the importance of health systems evidence, availability of qualified health systems researchers, communication and networking between policymakers and researchers, wide dissemination of research, and qualified policymakers (Table 6).

Respondents most frequently suggested building the capacity of policymakers in locating proper information and assessing the quality of research, its cost effectiveness and local applicability (13%). Increasing funding and investments in health research, improving dissemination and translation of research, and conducting relevant health systems research to inform policy decisions were also commonly mentioned suggestions for improving the use of research in policy. Other commonly mentioned suggestions include establishing evidence- to- policy decision support units to support policymakers in using research in policy, conducting sensitization and awareness workshops on evidence- informed policymaking, and enhancing contact and exchange between policymakers and researchers through meetings and conferences and setting easy and effective methods for communication and networking (Table 6).

### Policymakers’ training needs

Policymakers frequently listed the need for training on how to develop health policies and use evidence in policymaking. Additional training areas include; training on policy analysis, health economics, setting policy-relevant research priorities, strategic planning, conducting operational health systems research, budgeting and financial management, and general management and leadership.

## Discussion

There is very limited empirical work on evidence- informed health policy and KT across multiple countries in the Middle East region. By exploring policymakers’ views and practices regarding the use of health systems evidence in 10 countries from the Middle East and assessing contextual influences in health policymaking, this study is an effort to provide essential knowledge that can be used to design, promote and sustain effective KT strategies that would strengthen health policymaking in the region.

Policymaking is a complex process and our findings show that a myriad of factors influence health policymaking and often compete with health systems evidence. Our findings show that factors such as values of governing parties, corruption, and weak administrative structure are among the many factors that play a major role in the health policymaking process in the region. Most countries in the region fare poorly with corruption and democracy indices, reflecting increased possibility for policy formulation based on personal preference of government actors rather than evidence [30-32]. Literature also argues that the existing political governance or the ruling ideology in a given country can affect the application of evidence [33]. Furthermore, the influence of the political context and a corrupt and unstable political environment hinder the use of evidence in policymaking, while pressure from stakeholders and public opinion results in a culture conducive to the use of research [9,19,34]. For instance, in Tunisia, values of governing parties played a major role in the policymaking process. The index for voice and accountability in Tunisia, which captures the extent to which citizens are able to participate in selecting their government, is lower than that of the region and of other LMICs [35], indicating that the political regime rather than public opinion exerts a strong influence on policymaking. On the other hand, public opinion was stronger in Bahrain and weaker in Pakistan than in other countries. Furthermore, the impact of the media was stronger in Jordan and more minimal in Palestine compared to other countries. This is further supported by a high media freedom index in Jordan and a low media freedom in Palestine [36].

The influence of private providers, insurers, and professional associations was more pronounced in some countries more than in others. For example, private health providers and professional associations were more influential in Lebanon, where they are the main providers of health services [37]. However, in Yemen the role of nursing associations was weaker than in other countries and private insurers exerted a strong influence on policymaking. It is important to bear in mind that both Lebanon and Yemen have pluralistic health systems and are classified as weak states in the sense that private sector has an upper hand.

Findings show that limited health funding also competes with the use of research in the region. Resource constraints, in terms of both human and fiscal resources, were found to limit research utilization in LMICs [19]. Moreover, lack of coordination across various stakeholders also plays a major role in health policymaking in the region and was shown in the literature to decrease the use of evidence into policy [9]. It was interesting to note that countries of lower income status such as Yemen and Jordan reported greater challenges with lack of coordination across different stakeholders.

Our study shows that donor organizations exert a strong influence on health policymaking processes in the region. They can facilitate the use of evidence in developing countries; however, they may dictate their agendas on recipient countries from the region and hinder evidence- informed policies [9,38]. It is therefore crucial that countries work with donor agencies to ensure that research is translated into evidence and is of value to policymakers in developing health policies.

Given the diversity of the policymakers who were surveyed in this study and the different contexts in which they worked, it is striking to find that common influencing factors were identified. Policymakers outside the government play an important role in the policymaking process and may influence the policymaking process. This may be due to the fact that policymakers outside the government are often more affected by decisions made and thus they try their best to influence the policymaking process to influence the decisions being made to their advantage [39]. This being said, it is also important to note that there are key contextual factors that are different in terms of their influences in these countries. These differences in context would suggest that different strategies can be devised for countries to translate health systems evidence into policy [1]. Understanding the context in which users of evidence operate in provides valuable insights into effective methods to support KT [19].

Our findings show that policymakers recognize the benefits of integrating health systems evidence in the policymaking process. Policymakers from the region use globally produced evidence in formulating health policies such as vaccination schedules and accreditation strategies. However, policymakers did not use evidence when other contextual forces including politics competed with evidence such as in the reported examples related to establishing new healthcare centers and determining health human resources strategies. This means that even when relevant research exists, it can be overlooked due to other factors and constraints that could be more relevant for the decision making process and that different contexts can lead to different utilization of the same evidence. While global knowledge on certain areas is available, study findings also show that there is a need for locally produced context- specific evidence to formulate policies on the distribution of healthcare workers as well as the application of chronic diseases and accreditation programs. As literature indicates, contextualizing evidence for effective policymaking is a key challenge for health systems [40].

Findings from this study corroborate those previously reported from semi-structured interviews conducted with health care managers and policymakers from Canada and the UK [10] and a survey of policymakers and researchers was in Australia [2] as well as interviews with policymakers from LMICs [19]. Similar to findings from the region, policymakers from Canada and the UK also pointed out that many factors other than research evidence influenced policymaking, including financial sustainability, local competition, pressure from stakeholders and public opinion [10]. Furthermore, the use of research evidence in policymaking processes, as reported by policymakers, is comparable to those from Canada, the UK [10], and Australia [2], whereby the majority reported using evidence to inform policy content. While policymakers from Australia and LMICs placed high value on the use of research evidence in policymaking [2,19], findings from this study indicated that limited value given to research hindered evidence- informed policymaking. The lack in policymakers’ technical capacity to access and use research evidence, the need for more contact and exchange between researchers and policymakers as well as for more policy- relevant research was also emphasized by policymakers in previous studies [2,19].

### Strengths and limitations

Our study has three main strengths: (1) it is among the very few studies (if not the first) to explore the views and practices of policymakers in 10 countries in the EMR on the use of health systems evidence including contextual influences in policymaking; (2) we sampled a very diverse group of policymakers, including governmental entities, professional associations, private sector and civil society representatives, donor agencies across the ten countries; and (3) we used combined quantitative and qualitative research data analysis methods. Our study has a few limitations. It presents results on the use of evidence in policy based on the perception of a purposefully selected sample of policymakers. Hence, findings might not be representative of all policymakers in the region and results may not be generalizable across the region. This study was not intended to include a representative sample since it is difficult to obtain given the mix of policymakers and the diversity of contexts in which health policy decision- making occurs. It is worth noting that there was a variable response between different categories of policymakers within each country whereby the largest group of respondents in most countries were policymakers affiliated with the MOH. Moreover, there was a variable response rate across countries (range: 9–42 respondents per country). The varying degree in responses in some countries may be explained by the fact that more positions in the sampling frame are filled in some countries compared to others. This may be due to larger health systems including reform initiatives, larger populations, etc. For example, in Pakistan 13 positions were filled for national NGOs compared to two in most countries. In Yemen, eight positions were filled by managers of pharmacists’ associations compared to one in other countries. Despite this, we achieved an average of 23.7 respondents. Furthermore, given the nature of policymakers’ work (i.e. busy time schedule, last minute meetings, etc), the response rate of 56.3% obtained in this study is considered acceptable. The response rate is also comparable to response rates on surveying policymakers, stakeholders, and researchers from the region: researchers from the region: 56% [41], 64.6% [28]*,* and 83 policymakers from six countries [19].

In addition to the above, it was difficult to discuss the different contextual factors that determine country specific results within the data available. This may be due to the fact that context mapping has not yet been conducted in the region to identify specific factors and indicators that reflect use of evidence in policymaking. Country specific factors may have had a major role in determining the findings in the qualitative survey component and the section on comparing responses by study country. Despite the fact that such factors are important, discussing them is beyond the context of this specific paper. Discussing the context-specific issues that govern how policies are made and how evidence is used should be given justice by making them the focus of future country case studies that include additional data on the policymaking environment of study countries.

Another limitation of this study is the social desirability bias inherent with self- reported questionnaires. Respondents may have provided the answers they considered desirable by the investigators. Responses may present either true beliefs or perceptions of what respondents thought researchers wanted to hear, or a combination of both. However, it can be safely assumed that the results are not overly inflated because of the positive nature of almost all the questions. Furthermore, self- reports of current behavior provide clear information on where improvements should be implemented [42].

### Potential strategies to improve the use of research evidence

In order to strengthen the use of evidence in policies, efforts should be targeted to promote a supportive climate for evidence- informed policies, produce, package, and disseminate evidence, facilitate users’ pull, and promote exchange between researchers and policymakers [6]. Based on study findings, potential strategies for increasing the use of health systems evidence into health policies at each of these levels in the EMR are proposed.

#### Increasing funding and investments to support evidence to policy activities

Policymakers from the region suggested increasing health research funding to improve evidence- informed health policymaking. Improving research infrastructure and funding is important to generate policy- relevant evidence for policymaking [2]. Several activities can be employed in this regard, such as building the organizational culture for health systems research and providing incentives and resources for policymakers and researchers to engage in evidence- to- policy activities [2,19]. In a study about KT in developing countries, it was found that the application of research might be hindered when there is a lack of financial resources, while pressure from stakeholders and peers, and a climate conducive to the use of research can contribute to the success of KT activities [22].

#### Producing policy- relevant research evidence and improving packaging and dissemination of research

Study findings show that there is a need to enhance the production of policy- relevant research and to improve the ways with which researchers present and disseminate evidence to policymakers. In order to increase the relevance of research to policy, it is important that policymakers identify and communicate gaps in knowledge and policy priorities for research to researchers [2]. Furthermore, researchers should enhance their understanding of the policymaking context, focus on policy- relevant concerns, and improve the presentation of research results and implications [2]. The production of systematic reviews that address policymakers’ policy priorities is an important KT strategy that can be employed in the region. A recent regional study identified policy relevant research priorities for health financing, human resources for health and the role of the non-state sector. So far, little progress has been made in generating evidence on these priority areas [3].

Policymakers from the region also indicated that the available research was not delivered at the right time and that it lacked information on its quality and local applicability and actionable messages. Literature also shows that timeliness and relevance of research and the inclusion of a summary with clear recommendations facilitate the use of evidence in policymaking [9,10,19]. The establishment of clearinghouse mechanisms, evidence to policy support units, national databases, or institutional mechanisms in academic institutions or at Ministries of Health were also suggested to improve research packaging and targeted dissemination to policymakers in the region.

#### Building the technical capacity of policymakers

It was discussed earlier that policymakers’ lack of training and awareness on using evidence was a major barrier to using evidence in health policymaking in the region. It is worth noting that there are no established training and education programs to date that target policymakers in the Middle East region. As such, many policymakers might not have the skills and the scientific, professional or policymaking background. Building the capacity of policymakers in the region to locate the proper information and assess the quality of research, its cost effectiveness and local applicability was suggested by respondents to improve evidence- informed policymaking. Literature shows that lack of policymakers’ skills and expertise decreased the prospects for research use and that training can affect their decision making and the quality of decisions taken [10]. Various studies support a greater emphasis on training of policymakers since such training would foster a more positive attitude towards the use of research findings and boost receptivity [43]. In countries where research and policy connections are strongest, some of the senior administrators have had research experience or interest as part of their prior education [43].

#### Increasing communication and exchange between policymakers and researchers

Findings clearly indicate that there is a need to improve communication and exchange between researchers and health policymakers in the region. In fact, less than half of the respondents (43.1%) indicated having close collaborative relationships with researchers*.* Two- way communication and exchange can facilitate a mutual understanding of policy questions and the kind of knowledge that is needed to answer these questions. It can also inform researchers on the decision making process and on what policymakers consider as timely, relevant, or good quality research [9]. Policy dialogue meetings, informal opportunities for interaction, joint workshops for policymakers and researchers, establishing fora for knowledge sharing, and actively involving policymakers at various stages of the research process are among the most important strategies for increasing the prospects of research use [2,9,10,19].

Literature also suggests that the interaction between researchers and policymakers and the existence of an accountable “receptor” function in the government would favor the use of health services research in policymaking [44].

Researchers and policymakers in the region should create more opportunities for interaction. Moreover, research funders need to emphasize that researchers should work in partnership with policymakers to ask and answer relevant policy concerns.

## Conclusion

Health policymakers in several Middle Eastern countries recognize the importance of using health systems evidence in health policymaking. Our findings show that there are no negative attitudes by policymakers toward research evidence, its use, and benefits in the policymaking process. Strengthening health policymaking by using effective KT strategies requires serious efforts by both policymakers and researchers. This study identified barriers and facilitators for the use of health systems evidence in policymaking and it is hoped that the findings would prompt policymakers and researchers to start a dialogue and cooperative relationships.

Our study shows that fostering evidence- informed policymaking requires a clear understanding of the national contexts in which policy decisions are made. The process through which evidence from health systems research is utilized in policymaking is complex and multidimensional. To make significant impact on policy decisions, one must reflect a deeper understanding of the context in which these decisions are made.

Future studies from the region should also focus on investigating how policymakers “use” or “not use” health systems evidence in relation to certain policy issues, and how evidence interacts with context. In addition, future research should focus on assessing policymaking organization capacity to acquire, assess, adapt and apply research evidence. Future studies could also focus on exploring the link between the use of evidence and health status indicators in a given country.

Lastly, our study findings are important and timely in light of the changes that are unfolding in some Arab countries. Findings can provide some insights in setting the agendas for the new regimes as they strive to strengthen health systems and policy. Also, they can provide a baseline to undertake an analysis of the underlying transformations and their respective health policy implications including the way evidence will be used in the future into policy decisions. Study findings can also help inform and direct future plans and activities for the EVIPNet EMR and Middle East and North Africa (MENA) Health Policy Forum, in addition to being useful for countries that host or are planning to host KT platforms in the region.

## Abbreviations

AUB, American University of Beirut; EMR, East Mediterranean region; EVIPNet EMR, Evidence Informed Policy Network- Eastern Mediterranean Region; IRB, Institutional review board; KT, Knowledge Translation; LMIC, Low and Middle Income Countries; MENA, Middle East and North Africa; MOH, Ministry of Health; NGO, Non-Governmental Organizations; SPSS, Statistical Package for Social Sciences; WHO, World Health Organization.

## Competing interests

The authors have no competing interests to declare.

## Author contributions

All authors meet criteria for authorship. All authors approved this version of the article for publication. FJ contributed to the conception, design, and interpretation of the data as well as to drafting and critically revising the article. JNL contributed to the conception, design and analysis of the study as well as critically revising the article. NA and DJ contributed to acquisition of data, analysis, interpretation as well as drafting and critically revising the article. WA assisted in study design in addition to customization and finalization of the tool. SR contributed to interpretation of data, and critically revising the article.

## Financial disclosure

This study was jointly funded by the Global Health Research Initiative and the World Health Organization – Eastern Mediterranean Regional Office. The funders had no role in study design, data collection and analysis, decision to publish, or preparation of the manuscript.

## Pre-publication history

The pre-publication history for this paper can be accessed here:

http://www.biomedcentral.com/1472-6963/12/200/prepub
